# Motivation, self-determination, and reflexivity of researchers in comedic public engagement

**DOI:** 10.1177/09636625241291464

**Published:** 2024-10-30

**Authors:** Áine Gallagher, Claudia Fracchiolla, Jessamyn A. Fairfield

**Affiliations:** University of Galway, Ireland; American Physical Society, USA; University of Galway, Ireland

**Keywords:** comedy, motivation, public understanding of science, science communication, self-determination

## Abstract

Understanding motivation and impact of participation in public engagement programs is crucial for fostering dialogue between researchers and the public. Using Self-Determination Theory and Reflexive Thematic Analysis, in this study we analyzed motivation and impact on identity of researchers participating in Bright Club Ireland, a public engagement project where academic researchers learn to use stand-up comedy as an informal and accessible means of communicating their research, then perform at a public-facing variety night alongside professional comedians. Through semi-structured interviews and focus groups, we found that participation in Bright Club is largely intrinsically motivated, driven by researchers’ desire to gain skills, be recognized as experts, and present their own perspectives on their disciplines. These findings shed light on how participation in public engagement can promote a sense of autonomy, competence, and relatedness among researchers, and highlight the role of creative expression in facilitating reflection and growth.

## 1. Introduction

Humor is one of the most potentially revolutionary approaches to breaking down barriers of access, engagement and equity between scientists and the public. Constructing humor is a way of casting light on the human condition (Watson, 2015), and laughter as a heuristic cue both heightens affective response of an audience and improves their engagement with content such as science (Cacciatore et al., 2020). Unlike more staid traditions of science communication, humor targets affective knowledge as well as cognitive knowledge, connecting new ideas to everyday experiences (Baselga et al., 2022). Humor can inspire solidarity (Scott, 2011) as well as divisions between groups (Chattoo and Feldman, 2020), and while good-natured humor can inspire change, disparaging or offensive humor can alienate audiences (Kaltenbacher and Drews, 2020). Therefore, the type of humor used is critical: in educational settings, positive humor can help aid in knowledge retention, collapse socioeconomic differences in performance, and challenge stereotypes about who can become a scientist (Banas et al., 2011; Chan and Udalagama, 2021; Heras et al., 2020). Stand-up comedy can be seen as a form of science communication that maintains a deficit approach (Riesch, 2015), though even dialogic and participatory engagement retains some deficit model elements (Metcalfe, 2019). However, it is a method where researchers take science to people, in a form that they see and enjoy in everyday life (Humm and Schrögel, 2020). The use of humor has been shown to lead to an increase in the perceived credibility of scientists communicating through comedy (Chan and Udalagama, 2021; Yeo et al., 2020a), as well as encouraging critical reflection on the part of both the audience and the performer, enabling discussions of science alongside the social and cultural contexts that it occupies—a frequently stated goal of science communication which is rarely achieved (Metcalfe, 2019).

Engaging the public with scientific research is particularly important because scientific knowledge occupies a central role in every global and societal issue that communities face today (Davies et al., 2021; Delaney and Tornasi, 2020). A number of international public engagement event networks have sprung up to create opportunities for early career researchers and build capacity for public engagement, including: Bright Club, a comedy night where researchers receive training and perform comedy to discuss their work (Roche et al., 2020); Famelab, a science communication contest where participants present their research in three minutes to the public (Zarkadakis, 2010); and Pint Of Science, where researchers provide public talks about research in local pubs (Paul and Motskin, 2016). The reported benefits of these initiatives include capacity building for communication skills among early career scientists (Zarkadakis, 2010), enhancing the public’s understanding of scientific research and having a lasting positive impact on the careers of the young scientists who take part (Paul and Motskin, 2016), and strengthening of discipline-based and intersectional identities (Fracchiolla et al., 2020; Mullen et al., 2020). Critical analysis of public engagement activities has highlighted the emphasis on the descriptive methods of “how” to do public engagement within literature (Stilgoe et al., 2014), with evaluation more commonly focusing on the best ways to engage the public, rather than the best ways to engage researchers and the impact that this engagement can have in dialogue-driven iterative design of public engagement programs (Izadi et al., 2022; Poliakoff and Webb, 2007).

The factors that motivate researchers to take part in public engagement are critical to understand in order to improve the uptake and efficacy of public engagement activities. A national survey in the US, using a cross sectional sample of biomedical scientists, found that higher career status, organizational autonomy, and a sense of self-efficacy contributed to scientist participation in public engagement activities (Dudo, 2012). Similarly, Besley et al. (2012) show that perceived efficacy and positive attitudes toward public engagement are the most likely factors to predict participation. Gender and race have also been identified as important factors for participation, as underrepresented groups are more likely to volunteer their time for informal public engagement activities to serve as role models (Collins, 1990; Williams, 2018). However, in a study of contract researchers, many barriers such as lack of autonomy were identified by those with an interest in doing more public engagement (Davies, 2013). Critical questions of equity in public engagement are too often reduced to questions of access, where less hierarchical and more subversive approaches are needed to challenge the existing and often unjust structures of science (Philip and Azevedo, 2017). Dudo (2012) highlights the number of complex factors that can affect a person’s attitude toward public engagement, with factors such as previous experience, belief in the importance of public engagement, and perception that colleagues are participating all contributing to the decision to take part (Foster et al., 2010; Fracchiolla et al., 2016, 2020; Prefontaine et al., 2021). These complexities indicate that a deeper theoretical framework for motivation to participate in public engagement is needed. Furthermore, different models of public engagement may be attractive to different researchers, and treating public engagement as a monolith may be insufficient if we wish to understand what factors in different programs appeal to the individuals who take part.

Here we focus on one model of public engagement, Bright Club Ireland, and review the experiences of researchers who have taken part. The Bright Club model prepares researchers to communicate their work using stand-up comedy at public facing events, across disciplinary boundaries, by seeking out surprising and compelling stories from their professional lives (Roche et al., 2020). Bright Club Ireland events are centrally coordinated by a small team, providing comedy training for academic researchers and producing events which also feature professional comedians as MCs, performers, and headliners in cities and festivals across the country. These events are not solely focused on scientific research and communication, and hence capture a broader audience compared to events with “science” in the name. While humor has been shown to improve engagement for audiences of science communication (Yeo et al., 2020b), it stands to reason that it will affect practitioners as well. The training uses improvisation exercises, which have been used to teach science communication in a university context (Ponzio et al., 2018; Rossing and Hoffmann-Longtin, 2016), and shown to improve self-efficacy in women and empathy in men (O’Connell et al., 2020) which are both important factors in science communication. The Bright Club training also gives an opportunity to workshop speaker talks, providing practice, feedback, and a sense of community. Using this unique event format, we present an in-depth analysis of why people may choose to engage in this form of public engagement, to answer the research questions:

1. What motivates participants to take part in Bright Club?
2. What impact does participation in Bright Club have on participants’ professional, personal or discipline-based identity?

## 2. Theoretical background

To understand the motivation of academics and researchers to participate in public engagement activities and how this may have an impact on their professional lives requires motivational theories that connect to identity. Motivation is the process that initiates behaviors, based on the satisfaction of fundamental psychological needs. In the Theory of Planned Behavior (Ajzen, 1991), past behavior and a person’s perceived behavioral control, as well as descriptive norms, can indicate future behavior. Poliakoff and Webb (2007) found that past behavior and perceived ability, as well as the perception that others in one’s social context also participate, were key factors predicting scientist participation in public engagement.

Previous research has shown that extrinsic rewards do not make scientists more likely to engage in public engagement activities (Dudo, 2012). Studies have also looked at the impact of perceived control over individuals’ intrinsic motivation (Deci, 1975; Lepper and Greene, 1975) explaining that the motivation changes when a person’s perception of why she is doing the activity changes (Deci and Cascio, 1972). For a person to truly be intrinsically motivated she needs to be free of external pressures (Deci, 1980), and external work factors such as supervision, status and salary do not motivate individuals and can even produce negative effects over the long term (Acquah et al., 2021). Therefore, internal motivational factors must be of paramount importance.

Therefore, we elected to use a framework proposed by Deci and Ryan (1991) for studying motivation based on three need constructs: autonomy, competence and relatedness. Autonomy is generally defined as self-governance, and in theories of motivation, autonomy is seen as the individual’s perceived control over engagement in a task (Pintrich and Schunk, 1996). Competence refers to the desire to achieve mastery and feel accomplished in interactions with the environment (Harter, 1978; White, 1959). Relatedness has been defined as the desire to belong to a group or community (Baumeister & Leary, 1995; Reis, 1994). Individuals’ instinctive needs act as the foundation for personality integration and self-motivation, and when these needs are satisfied, individuals will be more self-motivated and experience well-being (Ryan and Deci, 2000; Ryan et al., 1995).

Self-Determination Theory (SDT) suggests that the satisfaction of the three basic psychological needs is essential for an individual to perform at their optimal potential and to feel greater enjoyment, satisfaction and psychological adjustment (Deci and Ryan, 1991). Conditions that support an individual’s experience of autonomy, competence, and relatedness can cultivate high motivation and engagement, which translates into a person’s enhanced performance (Deci and Ryan, 2000). Therefore, SDT can be used as a framework to study the impact of social and cultural factors on people’s satisfaction of those psychological needs, and hence on their motivation to engage in different tasks, activities, and/or communities (Ryan and Deci, 2000). SDT has also been used to understand motivation for enjoyment of narrative and interactive media (Rigby and Ryan, 2017), implying it is a meaningful lens for analyzing the creation of comedic material at an event like Bright Club.

## 3. Methodology

This study was qualitative in nature and data were collected using a semi-structured interview protocol, with one focus group of five participants and four individual interviews. Both group and individual interviews were used to develop a richer exploration of participant motivation (Lambert & Loiselle, 2008). The group interview took place first, with the semi-structured nature chosen to allow for a natural discussion to capture participant responses, while also prompting additional discussion. Following an initial analysis of these data, individual interviews with different participants were held to triangulate the focus group data and deepen results. The same protocol was followed for individual interviews, with questions focused on why participants decided to take part in Bright Club, what their expectations were of taking part, and the perceived effect that participation had on themselves and others. Audio recordings were taken during each interview, and transcriptions completed by an external contractor. Interview protocols were agreed upon by the researchers in advance; the full set of questions is listed in the Supplemental Material.

### Recruitment

Convenience sampling was used to recruit participants for both the group and individual interviews within this study. A total of 26 participants were invited to participate in the group interview via email, all of whom had taken part in Bright Club Ireland since the beginning of 2018. The training and production approach prior to 2018 had not yet been standardized, therefore participants from this timeframe would not have been a comparable group. In total, five participants agreed to take part in the group interview, which took place in 2019. Participants were aged between 25 and 45, and two were female and three were male. All participants came from different disciplines including electrical engineering, geoscience, psychology, medicine, and ecology. Three participants were postdoctoral researchers, one had a master’s degree and one was an assistant professor. Most participants had only participated in Bright Club once, and while some had other public engagement experience, for some Bright Club was their first entry into such an event.

For the individual interviews, five individuals were contacted, comprising all academics who spoke at the first two Bright Club events of 2022, and four agreed to take part in interviews. The gap in data collection was due to the pandemic, which shifted Bright Club events online for two years; the differing experience between in-person and virtual events was judged to be an extra variable in the speaker experience. Participants were aged between 35 and 45, and came from disciplines including chemistry, citizen science, immunology, and botany. Two were assistant professors, one was a postdoctoral researcher, and one was a lecturer. Three participants had taken part in Bright Club once and one participant had performed three times.

All participants received the same training prior to speaking at Bright Club, and all spoke at in-person events. All participants from both the group and individual interviews gave their verbal consent prior to the interview process. The confidentiality of the data has been preserved, and pseudonyms have been used to replace the names of all participants in this paper to protect anonymity.

### Analysis

Reflexive thematic analysis (RTA) is a qualitative method that involves iterative steps of familiarization with the data, coding, generating initial themes, reviewing and developing themes, refining, defining and naming themes, and writing up (Braun and Clarke, 2022). RTA was chosen to interpret the data in this study for a number of reasons: it offers a flexible approach for researchers, it recognizes that deductive and inductive methods for analysis are not mutually exclusive, and the subjectivity of the researcher is seen as a strength and necessary tool within the research and analysis process (Braun and Clarke, 2022). Initial data analysis was completed using MaxQDA software, and preliminary codes incorporated both deductive and inductive themes. Deductive themes refer to those where the analysis is directly shaped by existing theoretical constructs, and inductive themes are constructed as researchers analyze the data without previous consideration or constraint (Braun and Clarke, 2022). Our deductive themes were the three core components of SDT—autonomy, competence and relatedness—contextualized to the Bright Club environment. In Table 1, the definition of the constructs is accompanied by the key statements used to identify instances in which the statement should be coded with the particular component and some representative examples. Following this initial data analysis, the next phase of RTA was then implemented as the researcher re-engaged with the entire data set and all of the coded extracts. Codes and themes were hence re-examined by the researcher and the final themes were conceptualized from the results based on the research questions. The full list of codes is available in the Supplemental Material.

**Table 1.** Definitions, key statements, and specific examples for the three factors of SDT. The key statements are taken from the validated survey of SDT (Deci and Ryan, 2000).

SDT constructs	Key statements	Examples
**Competence** Desire to or a sense of having developed skills, having a sense of accomplishment and having increased insight into one’s own capabilities in terms of professional knowledge and identity.	• I was able to learn interesting new skills • I did not feel very capable • I do not feel very competent at what I do • I felt pressured • People told me I was good at what I was doing • I felt a sense of accomplishment from what I did • I do not get many opportunities to show how capable I am	“it really gave me a lot of, not confidence, but like relaxed me. Because I had a few other like interviews, or meetings kind of and because I’d done the Bright Club, like I, the stress level before this was so much lower, I was like okay I’ve done Bright Club whatever” “In terms of kind of as I said general public engagement thing just to, it’s something you have to practice as well. So it’s kind of any way of building that skill set and yea. It’s a good way of doing that I think.” “People come talk to you like after the, after you did it. But because I have the most interesting conversations, like oh I liked this and that.” “Yea like I went very much towards the side of the, what the practical implications of my work are. So kind of spent a bit of time explaining it and then kind of brought everyone back to why it matters to them. I think people remember that more so than why you’re actually talking about, in terms of your own work.”
**Autonomy** Defined as an internal drive and desire for participation that is connected to personal choice, personal preference, personal expression and a sense of control.	• I feel free to express my ideas and opinions • I have to do what I am told • People I interact with take my feelings into consideration • I feel like I can be myself • There is not much opportunity for me to decide for myself how to do things	“For me like my expectation was I could do a piece that I could actually say what was on my mind. Because my subjects I deal with are so kind of sensitive and stuff. It was an opportunity to say like not to be PG13 and to talk about all that.” “I was very deliberate not to have any puns in my set. . .Because I don’t know why but geology and puns is just. . .I was not going in there, so. . .There’s more to us than puns.” “I didn’t want to bring the culture of my discipline or sense of humour, I don’t like engineering jokes, like two vectors going for a stroll (All laugh). Or the secret, or convolutions or whatever, I don’t find them funny.” “So I actually thought that the process of writing actually helped me kind of you know, break through the wall of like, oh I need to kind of you know, please someone or something like this. But actually [it] helped me expose my own humour.”
**Relatedness** Defined as the nature and the effect of interactions with others in their social contexts and the desire to be a part of and belong within these environments.	• I got along with people I talked to • I considered the people I interacted with to be my friends • I got to positively interact with peers • People were generally pretty friendly toward me • I did not get to interact with people I am close to • I kept to myself and did not have a lot of social contact • People I interacted with care about me • People I interacted with do not seem to like me much	“It wasn’t something I would have electively done myself had I not been encouraged to do so by my peers.” “It’s something that we’re kind of strongly encouraged to do in my centre.” “Yea we’re the same, yea we’ve had very strong kind of public, so I’ve worked in a few different labs. And they all have very strong public engagements over the Bright Club in particular, my PI, my boss at the time had previously taken part so I had a kind of a framework to look at.”

### Limitations

While the group interview and individual interviews were detailed, each running over an hour, the sample size is relatively small. In addition, the focus group and individual interviews took place three years apart, and although the Bright Club training and event production approach were standardized by this time, it is possible that this contributed to some variation between participant experiences. The group interview was completed by two researchers, one of whom was also in the position of Bright Club Coordinator at the time. This same researcher completed individual interviews with respondents. This made for a candid and informal interview environment, as the coordinator was already deeply familiar with the program and some of the participants, but could have also led to some bias within the nature of the questions and responses given. The results are situated within the Bright Club Ireland context, and varying the execution of a project using comedy for public engagement (for example, removing the training element or using video instead of live performance) could vary the participant experiences, and thus the impact on participants.

Another important limitation for this research project is that we cannot determine causation, only report participants’ semantic descriptions of their interaction with the environment and their perceptions of how that interaction makes them feel. Finally, there may be other variables that were not considered which could affect participants’ motivation and therefore behavior.

## 4. Results

### What motivates people to take part?

In general, the themes identified in the focus group and the individual interviews triangulated each other well and fit within the SDT framework. Perhaps due to the unique nature of Bright Club as a public engagement platform, an additional theme of creativity and self-expression was observed, which also seemed to provoke reflection on the part of participants.

#### Autonomy

Intrinsic motivating factors centered around autonomy and competence were the most common reasons, mentioned by all participants, for taking part in Bright Club. Mark commented:

“I had those moments when I’m like bored with my life. . .and I’m looking for crazy things to do. And so I saw Bright Club and was like. . . oh yeah, let’s sign up.”

He was interested in the challenge presented by Bright Club and his interest emerged from a personal desire to have a new and exciting experience. Roberta reported her interest in how storytelling may encourage a more accessible perspective on science particularly:

“People generally don’t. . . think about art in a way that’s inaccessible. They say I don’t like this, you know, and I feel like people should be able to do something similar with science. . . I think comedy kind of opens that up. . . because it allows people to explore what they like.”

Half of the participants interviewed had a personal interest in comedy, which added to their motivation to participate, as stated by Emily:

“I really love comedy and I thought it sounded like something a little bit different and a little bit fun.”

The desire to explore something new in their public engagement may have stemmed from the desire to discover that motivates many scientists in their research careers.

#### Competence

Those with an apparent innate value in public engagement, two thirds of participants, described their participation as an opportunity to develop enhanced skills and methods to share science with the public and influence others. As Julie stated,

“I’ve always been interested in public engagement. And it’s kind of a skill, communicating my research in a new and fresh way is something that I’ve always been interested in. . . Bright Club was very much a challenge, to do that in a very different way to any way I would’ve done previously.”

Emily described her desire to take part so that she could positively influence her students.

“I’m constantly trying to encourage my, particularly my graduate students to do outreach, to do conference talks. . . I thought it would be good to have an example to say to them, ‘Look, I was terrified doing this. This is not my comfort zone.’”

Roberta talked about her existing interest in public engagement and her desire to do something new, connecting to both competence and autonomy:

“Yeah, a lot. . .So I’ve done like a lot. I’ve made a board game. (Laughs). Loads of talks, loads of school talks. . .So I think in that sense like my experience was a bit different because I wasn’t starting from very little. It was like, yeah, like an extension of what I was doing but to a place where I’d never been before.”

Skill development, whether via Bright Club or other public engagement initiatives, can be seen as furthering the civic values which often drive participation to begin with.

#### Relatedness

Influence from peers and social networks emerged as the most common extrinsic motivating factor, mentioned by half of participants, that prompted people to volunteer for Bright Club. Participants stated:

“It had been recommended to me by a friend/family member. It’d been on my mind for a bit. So it was the idea that she thought I could do this. So I thought I should give it a go and see.” (Igor)

“My initial interest was I saw somebody on Twitter had retweeted it, another academic.” (Ben)

However, intrinsic values in public engagement could also lead to tension with participants’ professional environments.

“We have a departmental outreach committee of which. . . it consists of me.” (Ben)

“I think in terms of my experience with [public engagement], it’s been very self-driven. I haven’t had anyone really come and say, you know, ‘Have you thought about doing public engagement, you know, what are you going to do? What’s going to be next?’” (Emily)

As discussion evolved on this point within the group interview, participants reported different experiences.

“Yea our research centre is, has some very strong public engagement programmes. We’re all very strongly encouraged to do things like this.” (Jim)

“But as an academic in a university generally it’s not something that you would get any extra credit for doing. It’s something that you do more so out of your own interest and your own time.” (Julie)

Jim responded to say that encouragement may be more directly targeted toward junior level staff.

“I would say like career level might be a thing there. Because it would be, within our group it would be much more aimed at PhD’s, post doc’s, whereas senior people are kind of left to their own things.”

Relatedness is clearly a key aspect of motivation for participation, which can be positive or negative depending on career stage, professional environment, and other context.

#### Creative self-expression

The final theme that discussions of participant motivation identified was the idea that the format of Bright Club felt right for them personally. Participants liked the opportunity to communicate in a way that expressed their own personality, as Igor explained:

“With Bright Club it was up to me what I wanted to talk about you know and what message I wanted to get out. . . And I guess maybe in a more (individual’s name) manner, in a way that is more akin to how I communicate.”

Participants also mentioned other public engagement platforms and outlined why these opportunities did not appeal to them:

“And also like I’m a little bit older. Like (science communication competition) tends to be for very early career people.” (Roberta)

“I’ve seen some of the competition, so like the (science communication competition) and so on. All these things and they just don’t work for me. I don’t see the point of competing on the science communications.” (Mark)

The sense that some public engagement had not only a specific type of audience, but a specific type of practitioner, was apparently a deterrent whereas Bright Club was perceived as allowing the participants freedom beyond the boundaries of their professional roles or traditional styles of science communication.

### What impact does taking part have for participants?

Participants reported direct professional gains and indirect personal impacts following their participation in Bright Club, which frequently connected to more than one theme. The direct professional impacts reported included having enhanced communication skills (competence) and a new sense of comfort and confidence (autonomy) when engaging in other forms of public engagement. Julie stated:

“[I]t’s given me a little bit more freedom around. . . how I communicate things and talk about them. . . A little bit more relaxed maybe.”

Similarly, Mark discussed how he now finds various forms of public speaking less stressful following his participation, indicating increasing competence.

“[Bright Club] was at this level of difficulty that it really gave me a lot of, not confidence, but like relaxed me. Because I had a few other like interviews, or meetings. . . and because I’d done [Bright Club]. . . the stress level before this was so much lower.”

Nearly all participants reported that they had gained a sense of their own expertise from taking part as well as greater esteem by taking part, touching on both competence and relatedness:

“I think certain people; you know professionally, kind of you know it got their attention. . . It kind of gave me a sense of being an expert, kind of establishing my reputation a bit more.” (Igor)

All participants reported impacts that clustered around relatedness, such as having a positive influence with their colleagues and peers to take part in public engagement, as Mark reported:

“It definitely raised awareness. . . to some of the people who wouldn’t necessarily be like very inclined to do public speaking, or public engagement. They thought, oh okay he did it, so perhaps I can do it.”

Many of the indirect personal impacts stemmed from the opportunity for participants to exercise their artistic license, connecting autonomy, competence, and relatedness to creative self-expression. For some, they commented on the creative process and how it allowed them to resist their professional stereotypes. In relation to his identity as a geologist, Jim stated;

“I was very deliberate to not have any puns in my set. . .there’s more to us than puns.”

Mark also commented on how he had never found stereotypical engineering humor funny.

“I didn’t want to bring the culture of my discipline or sense of humour, I don’t like engineering jokes, like two vectors going for a stroll (All laugh). . . the thing that surprised me was I didn’t know it was going to involve so much self reflection about myself.”

He also described how this process provided him with a newfound knowledge of himself and his personal sense of humor.

“The process of writing actually helped me. . . break through the wall of needing to please someone or something like this. But actually help[ed] me expose my own humour.”

Half of participants interviewed reported that having this space for creative exploration allowed them to conceive new ideas and have fun.

“I drew online dating molecules. . . I’m not an artist at all. And so it kind of like, it gave a space for me to push myself a little bit out of the zone.” (Roberta)

Half of the interviewees also described how their participation in a comedic form of public engagement fostered a space where they could connect more authentically to their social networks about their work. Roberta reported that her participation had allowed for enhanced connections to be made with her family:

“[Bright Club] is one of the things that’s connected me most closely with my family for example and that’s been really valuable. . .I wasn’t expecting to have my dad kind of sharing this with all his mates.”

The theme of an enhanced sense of connectedness was also evident, specifically for Ben, who had commented on his feeling of disconnection within his professional context.

“I’d say the major thing was it was really good to know that I could get up and do something that wasn’t my day job in front of other people. . . and it was really nice to know that some of the things that I find funny, other people did as well.”

Participants in the focus group discussed how the creative process allowed them to discover more about their own capabilities. Mark described how the process of writing allowed him to understand his limitations in terms of the information he wanted to share.

“I set myself a goal that I will explain to people the concept of bandwidth and why it’s important. Because I thought it’s key that people get something out of it, something that’s scientific. And then I realized, and this was an interesting reflection, that I just, I’m not yet ready to explain that concept to a general public.”

Mark viewed this new insight as a positive impact rather than a negative one as he now had the confidence to question the true level of expert knowledge held by others in his field:

“Actually because of this, I realised that I don’t know a single person that can explain it. . .What I’ve done I feel like whenever I have conversations, informal conversations with people, professors and so on, I say explain bandwidth to me. And then there is like, no, (All laugh) I can’t explain that, you can’t explain bandwidth.”

On a related theme, a third of participants reported how observing and working with professional comedians provided the insight that despite their experience, they too experienced nerves. This perhaps surprising access to the community of professional comedians was described by Julie:

“And something that really surprised me on the night was how nervous the real comedians were. I was like, why are you nervous (All laugh). You’ve done this loads. But it was almost a bit reassuring then as somebody doing it for the first time.” (Julie)

Finally, three quarters of participants made reference to the informal environment and how the live audience, natural feedback and atmosphere made it an enjoyable and reassuring experience, with Sarah comparing it to a “warm hug.” Participants also commented on the importance of audience response:

“Well I think the audience enjoyed it. And that gives you a bit of a buzz.” (Igor)

“And I think that really enhances communication as well so that felt really good that, you know, there was the audience that were responding.” (Emily)

In addition to this, nearly all participants reported how much they simply enjoyed the experience and demonstrated enthusiasm to participate again, such as Ben:

“Yeah, actually I did ask if I could. I’ve got another story so I think I’m on in November. . .I really really enjoyed it.”

## 5. Discussion

### Autonomy, competence, and relatedness

In both group and individual interviews with Bright Club participants, they reported stronger motivation from internal as opposed to external factors. The desire for a new personal challenge was a strong motivational factor for many, supporting previous findings about science comedy in Pinto et al. (2015). Our participants also reported having engaged in various forms of public engagement prior to their participation in Bright Club. This may demonstrate an existing sense of competence in these types of activities for participants, who also reported a desire to develop their skills in this field in a novel way.

Participants also reported that specific aspects of Bright Club appealed to them, such as the opportunity to communicate in a personally relevant way, the lack of competition and an underlying interest in comedy. Our results demonstrate that participation in Bright Club was generally not motivated by professional gain, and in fact was strengthened by the non-competitive and non-directive nature of the training. A prior study examining the experiences of scientists and theater makers who engaged in collaborative projects found that an interest in theater, an interest to learn about theater and a curiosity about novel experiences were among the factors that made scientists want to participate (Dowell and Weitkamp, 2011). These findings align with the autonomy component of SDT, and reinforce findings that comedy and sketch writing as a means of discussing science were shown to increase autonomy and engagement of students (Heras et al., 2020). And as Dowell and Weitkamp (2011) argued, public engagement that connects to the personal interests of scientists may benefit from increased participation.

There is also a close relationship between a person’s perception of competence in a certain activity and that person’s intrinsic motivation to participate in such activity. The more people believe they are competent, the more intrinsically motivated they are to participate (Deci, 1980). This was evident for Bright Club participants, as a sense of self-efficacy and previous involvement played a key role in their motivation for Bright Club. Competence influences intrinsic motivation under two specific conditions: first, the activity must be challenging enough that the individual has an interest in participating (Shapira, 1976); second, the individual needs to believe that her competence is self-determined, not due to external factors (Deci, 1980). This aligns with the fact that for the Bright Club participants, their desire to enhance their skills and public engagement methods came from their own inherent value in public engagement. Many reported being self-motivated in this field, even though they received little direct departmental support. These data support the findings of Poliakoff and Webb (2007) who found that career recognition did not predict intentions, and it is the “civic scientist,” contributing for the sake of wider society compared to personal gain, who is more likely to participate in public engagement.

While autonomy and competence have been found to be more influential for intrinsic motivation (Ryan and Deci, 2000), research has shown that satisfaction of those two needs is only achieved if there is a supportive environment that provides a sense of security and confidence, which will make intrinsic motivation more likely to flourish (Deci and Ryan, 2000). Relatedness, the third component of SDT, defined the extrinsic motivating factors for Bright Club participants who also described the positive effects of their external environments on their motivation, such as receiving support from friends or colleagues or having seen others take part in Bright Club. Some participants reported a desire to find their communities, given that they felt a social disconnect within their professional contexts, and in the group interview participants supported each other’s statements to this effect. The influences of peers and supervisors can affect engagement (Fracchiolla et al., 2016), and a lack of departmental support for public engagement has been found to be a barrier to participation (Davies, 2013).

This work highlights the potential positive or negative impacts that an individual’s environment may have on their participation. Here the positive influence from researchers’ communities is amplified by strong existing sense in their own abilities and a strong will to participate. A sense of confidence has been found to be instrumental in motivating participants to take part in public engagement (Dudo, 2012), and a lack of perceived competence is a major barrier for participation (Davies, 2013). When examining the intrinsic and extrinsic motivating factors for scientists to participate in public engagement, it is clear that these components are intertwined in a complex web of determinants, with one factor having the potential to affect and influence the other.

### Reflection and growth through creativity

For many Bright Club participants, the opportunity to use their artistic judgment in the creative process went beyond autonomy, creating a space for personal exploration, personal reflection, and personal growth. Stilgoe et al. (2014) argued that we now need to question what public engagement achieves and move toward models that include dialogue, participation and reflective practice. Including performing arts in public engagement projects can stimulate this dialogue, not only between researchers and the public but also between researchers and other performers, such as the comedians in Bright Club. Our results have shown that the process of participation in public engagement driven by comedy, scaffolded with training and support, can provide this space for reflection.

Bright Club participants also described a change in their initial expectations which was facilitated by this creative process, reporting that it enabled them to learn and discover more about their own individual personalities, their personal senses of humor and their professional identities. Their participation created a space for meaningful connections within existing social networks where they could present themselves in an authentic way. The concept of changing expectations was also observed for scientists working with theater makers, who began expecting their role to be expert consultants, but by engaging with the critical perspective of the theater makers embraced a more dialogic process that led to true interdisciplinarity (Dowell and Weitkamp, 2011).

Results showed that the Bright Club process enabled participants to engage in personal reflection, gain a greater understanding of themselves, understand and be comfortable with their personal limitations and find a way to connect with others without necessarily needing to fit in to existing stereotypes or expectations from their respective communities. This suggests that participation facilitated these individuals to transcend “the great gap” as coined by Rowan (1998, p. 83), where an individual can move beyond completely relying on the opinions of others and toward personal empowerment and growth, leading to an internal rather than an external locus of control. The scaffolded Bright Club training, which begins with improvisational exercises and finally enables participants to write their own script, supports participants to move beyond the opinions of others into greater self-actualization as experts. Results from existing literature similarly indicate that participation in public engagement helps researchers feel validated in their work (Davies, 2013), strengthened in their professional identity (Fracchiolla et al., 2020; Mullen et al., 2020), and motivated to pursue further participation opportunities in other public engagement programs (Fracchiolla et al., 2016). In public engagement programs combining science with dance, music, or engagement in informal settings, the ability to integrate one’s full identity with one’s disciplinary expertise was a prominent driver of participation (Mullen et al., 2020).

One simple but significant finding within our results was that participants enjoyed their Bright Club experience, they reported that it had been fun, that they were surprised by the level of positive feedback and one participant even compared it to a “warm hug.” Research suggests that spaces for engagement and reflection may contribute to a more motivated and confident scientific community (Fracchiolla et al., 2020; Mullen et al., 2020). Indeed for our participants, many had either performed in Bright Club more than once or had scheduled another performance, indicating that positive experience, for whatever subjective reason, may motivate further engagement. Several studies (Deci, 1971; Deci and Cascio, 1972) have examined the effects of positive feedback and recognition from peers on a person’s intrinsic motivation. They show that positive feedback satisfies the need for feeling competence, which in turn enhances intrinsic motivation (Deci, 1971,^1980^; White, 1959).

It is likely that the specific humor component of Bright Club contributed to this enjoyment. Previous research findings about laughter making science more engaging, laughter heightening affective response, and comedy as a way to challenge stereotypes and myths about who can be a scientist would all affect speakers alongside audience members (Cacciatore et al., 2020; Chan & Udalagama, 2021; Heras et al., 2020). Indeed, a joyful experience in public engagement is a great enticement to do it again (Besley et al., 2018; Fracchiolla et al., 2016; Gross, 2015), and helps ease the discomfort of growth.

While positive feedback is likely to contribute to a person’s sense of competence and motivation, results indicate that it is the opportunity for growth that people may also find most valuable from these activities. Deci and Ryan (2000) argue that environments providing autonomy, competence, and relatedness lead to engagement and improvement. Among the indirect and unexpected impacts reported by Bright Club participants was an enhanced sense of comfort in their own capabilities and limitations and a stronger sense of self-actualization, where they felt less confined by the opinions of others or the need to fit in. As Dowell and Weitkamp (2011) demonstrated, their participants became more comfortable with the concept of ambiguity and letting go of the need to be the “expert.”

Indeed, it is the indirect and potentially uncomfortable impacts of participation that appear to affect people’s individual sense of self, personal empowerment and self-actualization most strongly, and these seem to be higher for Bright Club than other similar public engagement projects due to the inclusion of comedy and space for self-reflection. The desire to engage and create must come from internal rather than external factors (Acquah et al., 2021; Dudo, 2012) and without this intrinsic motivation, the opportunity for growth is lost.

## 6. Conclusions

Understanding motivation for scientists to participate in public engagement, and measuring the impact of participation on these scientists, are essential to broadening both participation and the publics reached. In this study, we used individual interviews and a focus group to analyze motivation and impact for researchers participating in Bright Club Ireland, and found conceptual themes that were well-described by the constructs of self-determination theory (autonomy, competence, and relatedness), as well as a theme of reflection and growth through the creative process. Intrinsic motivation was key, expressed through the desire for a challenge, the desire for agency, and inherent values in public engagement. Besley et al. (2012) concluded that it is those who are intrinsically motivated who will find a way to overcome barriers. However they also note that consideration is needed regarding how to change institutional culture and potentially encourage a more positive attitude from a multi-tiered approach. For Bright Club Ireland participants, this internal drive was supported by extrinsic motivation in the form of both community support and finding new communities, which then enabled reflection and growth within the process of writing a comedy set. Participants in Bright Club found the development of comedic material, and the scaffolded training process, an important opportunity to reflect on their field, their own role within it, and their identity in the broader scientific community. We identified the presence of training in Bright Club as critical to these reflective modes, confirming recent work which showed that to induce real change in the audience’s attitudes, emotions, and knowledge about science, training is essential for public engagement events to help scientists reach broader audiences (Ocobock and Hawley, 2020). The humorous aspect of this specific form of public engagement likely contributed to both the sense of challenge, sense of reward, and overall enjoyment experienced by participants which has been found to mediate engagement and agency (Cacciatore et al., 2020; Heras et al., 2020).

While this is a case study, and the factors that appeal to Bright Club participants may not appeal to others, growing evidence shows that individual interest is an important motivator for public engagement participation, and diverse opportunities are required to meet the range of potential researcher interests (Dowell and Weitkamp, 2011). As Stilgoe et al. (2014) argued, in public engagement the public cannot be treated as a generic undifferentiated group, hence in recruitment of researchers to public engagement, researchers themselves cannot be treated as a single undifferentiated group. Especially in the context of the pandemic, which saw a reversion to deficit-led forms of public engagement (Roche et al., 2021), it is essential to develop new forms of public engagement that incorporate researchers’ full identities and affective responses alongside dialogue and participation, encouraging audiences as well to show up with their whole selves. Our findings here suggest we, as a community, should explore the indirect and messy impacts of public engagement, so that public engagement with science can be seen as a living and evolving organism and not a means to an end.

## Supplemental Material

sj-docx-1-pus-10.1177_09636625241291464 – Supplemental material for Motivation, self-determination, and reflexivity of researchers in comedic public engagement Supplemental material, sj-docx-1-pus-10.1177_09636625241291464 for Motivation, self-determination, and reflexivity of researchers in comedic public engagement by Áine Gallagher, Claudia Fracchiolla and Jessamyn A. Fairfield in Public Understanding of Science
