# Supplementary material for: Motivation, self-determination, and reflexivity of researchers in comedic public engagement
Source: Public Underst Sci. 2024 Oct 30;34(5):628–45. doi: 10.1177/09636625241291464 (PMC12177192; doi:10.1177/09636625241291464)
Supplement: sj-docx-1-pus-10.1177_09636625241291464 – Supplemental material for Motivation, self-determination, and reflexivity of researchers in comedic public engagement [file sj-docx-1-pus-10.1177_09636625241291464.docx]

**Motivation, Self-Determination, and Reflexivity of Researchers in Comedic Public Engagement**

Áine Gallagher^1^, Claudia Fracchiolla^2^, Jessamyn A. Fairfield^3^

^1^ Community Knowledge Initiative, University of Galway, Galway Ireland

^2^ American Physical Society, College Park, Maryland USA

^3^ School of Natural Sciences, University of Galway, Galway Ireland

# **Supplemental Material Contents**

# Supplemental Material 1: Directive Questions

Supplemental Material 2: Thematic Codes

# **Supplemental Material 1: Directive Questions**

Questions and subquestions related to Bright Club Ireland participation which were used in the focus group and interviews.

**Q_1 Why did you decide to do Bright Club?**

- - - Are there any **aspects** in particular **that attracted you** about BC?
    - Did you have any **hesitations?** If yes, what were they?
    - Has anyone done it more than once? Why?
    - Would you consider doing it again? Why?
    - Are there **reasons you would not do it again** in the future?

**Q_2 What were your expectations of taking part?**

- - - Did **you** **meet** those **expectations**?
    - Did the **Bright Club experience meet** your **expectations**?
    - Was there anything that surprised you about the experience? Can you give an example?

**Q_3 Do you think that Bright Club and your own participation has had an effect on:**

- - - the audience?
    - peers?
    - family/friends/local community?
    - How? In which way? Can you give an example?

**Q_4 Has participation had an effect on you?**

- - - How? In which way? Can you give an example?
    - Did you find **any particular challenges or surprises** through participation?
    - Did you **build or continue relations** with your BC peers after participation? (why/ or not?)
    - Include some scenarios/contextualize questions such as:
- In my experience etc. (e.g. group therapy / nice to meet people from different disciplines / imposter syndrome)

**Q_5 Have you participated in any other public engagement programs (prior or after Bright Club)?**

- - - What programs?
    - What were the differences/similarities?
    - Explore if preparation period is different given that BC is less specific in terms of guidelines
    - What aspects attracted you from each? Why?
    - In what ways do you think you’ve benefitted or gained from these experiences?
    - What is the most important thing you got out of them?
    - Have you noticed any changes in yourself after participation? Which? Examples?

**Q_6 How is public engagement perceived in your profession/discipline?**

- For you, is it a core element of what you do, and do you think you could be a ‘physicist for example’ if you did not participate in public engagement?

**Supplemental Material 2: Thematic Codes**

|  | Autonomy | Competence | Relatedness | Reflexive: Creative Self-Expression |
| --- | --- | --- | --- | --- |
| Motivation | Desire for Fun  PE Inherent Value  Defy Stereotypes / Be Themselves  Personal | Existing Beliefs  Strengthen Muscle New Challenges  Desire for Prof Gain  Lead Positive Image  Nourishment | Direct Professional Influence  Indirect Influence  Desire to find community | Naturally inclined – PE champions  Enjoyable and Fun  Comparing PE Platforms |
| Impact | Artistic License  Finding Personal Voice  Being Themselves | Learn Applied Skills  Things Are Easier  Nourishment  Gain Recognition  It's Hard Work | Feeling More Connected Socially  Positive Professional Influence  Context and Engagement  Learning from Others | Personal and Professional Identity  Understanding Capabilities |
